# Typhoon survivors' subjective wellbeing—A different view of responses to natural disaster

**DOI:** 10.1371/journal.pone.0184327

**Published:** 2017-09-06

**Authors:** Yaira Hamama-Raz, Yuval Palgi, Elazar Leshem, Menachem Ben-Ezra, Osnat Lavenda

**Affiliations:** 1 School of Social Work, Ariel University, Ariel, Israel; 2 Department of Gerontology and the Center for Research and Study of Aging, University of Haifa, Haifa, Israel; Stellenbosch University, SOUTH AFRICA

## Abstract

**Objective:**

Subjective well-being was evaluated three weeks after Super Typhoon Haiyan struck the Philippines. Based on the Conservation of Resources theory, which focuses on the role of resources in understanding adjustment following trauma, data was collected on lost resources. In line with the Conservation of Resources theory, four categories of resources were defined: objects—residential property; condition—gender health state and witness to injury; personal—coping strategies; energy–relationships.

**Design and settings:**

Eight hundred thirty-four people from the Philippines filled out self-report measures using an online interview system regarding: socio demographics data, subjective well-being, using the Delighted Terrible Faces Scale (DTS), disaster related experiences, coping strategies, personal relationships, obtained through support sources (close family, relatives and friends, community) and assessing problems with those relationships after Haiyan.

**Results:**

Subjective well-being was predicted by the following classes of resources: objects (home damage) condition (self-rated health and witness to injury), personal (positive reframing and self-blame coping strategies) and energy resources (relations and problems in relations).

**Conclusions:**

The results imply the important role individual’s resources (i.e. objects, personal characteristics, conditions, and energies) might play in promoting subjective well-being, following natural disaster.

## Introduction

On November 8, 2013, category 5 Super-typhoon Haiyan struck the Philippines, causing catastrophic damage throughout much of the islands of Leyte and leading to more than 6,000 fatalities across the country. This type of disaster might be considered a traumatic event in accord with the Diagnostic and Statistical Manual of Mental Disorders, 5th Edition (DSM-5), which defines trauma as "exposure to actual or threatened death, serious injury, or sexual violence” ([1], p. 271). Trauma can be experienced: directly, by witnessing the event, learning about the event, or through repeated or extreme exposure to aversive details of the traumatic event(s) [1].

A wide range of emotional, cognitive, biological and behavioral symptoms can follow in the aftermath of a traumatic incident [2].

Acute stress disorder (ASD) is one such posttraumatic stress reaction that occurs three days to four weeks after the event. According to the Diagnostic and Statistical Manual of Mental Disorders, 5th Edition (DSM-5), ASD includes at least nine symptoms from any of five categories (intrusion, negative mood, dissociation, avoidance, and arousal) [1]. To receive this diagnosis, the individual also has to display a reaction that causes significant distress or impairment in social, occupational, or other important areas of functioning. In addition, traumatic events include incidents in which individuals experience family separation and loss, individuals are injured or witness injuries or death of others and/or experience the breakdown of social support systems [3]. The up to date research on the utility of acute stress disorder diagnosis has found it to be highly predictive of post-traumatic stress disorder status, 3 to 6 months after the trauma. This finding was found valid either in cases with high likelihood of diagnosing post-traumatic stress disorder (i.e. full acute stress disorder criteria were met) or in cases with low likelihood of diagnosing post-traumatic stress disorder (i.e. at least two acute stress disorder criteria were not met) [4]. Thereby, the present study will focus on acute stress disorder.

In comparison to acute stress disorder, diagnostic criteria for the more commonly assessed posttraumatic stress disorder (PTSD) include a history of exposure to a traumatic event that meets specific stipulations and symptoms from each of four symptom clusters: intrusion, avoidance, negative alterations in cognitions and mood, and alterations in arousal and reactivity. A sixth criterion concerns duration of symptoms- at least six months after the trauma(s); A seventh criterion assesses functioning; and, an eighth criterion clarifies symptoms as not attributable to a substance or co-occurring medical condition [1]. This comparison between acute stress disorder and posttraumatic stress disorder highlights the importance of measuring acute stress disorder in order to prevent severe traumatic reactions, like posttraumatic stress disorder.

Over the last decade, there has been increased interest in strengthening positive emotions following adversity (e.g., [5,6]) indicating that increasing positivity can bolster problem-focused coping strategies [7, 8] reduce negativity and pathology [9] and decrease distress [10]. According to the broaden-and-build theory [5, 11] positive emotions are unique and adaptive because, at the crucial moment, they extend thought–action repertoires, and, over time with broadening, they build one's enduring physical, social, intellectual, and psychological resources [6]. Nevertheless, according to Mauss et al. [12] the pursuit of positive emotions might lead to maladaptive outcomes since it sets people up for disappointments resulting from unreachable expectations. In the current study, we aimed to evaluate subjective well-being and its link to loss of resources among persons living in the Philippines, following exposure to Typhoon Haiyan.

We used the term "resources", as defined in the Conservation of Resources theory [13]: “objects, personal characteristics, conditions, or energies that are valued in their own right or that are valued because they act as conduits to the achievement or protection of valued resources” ([13], p. 339). Objects relate to physical items of value due to their utility, rarity, or symbolism (housing, transportation); personal characteristics relate to traits that help with stress (coping, self-efficacy, resiliency); conditions relate to states of being that have value due to their general desirability (marriage, health state); and energies relate to resources that are valued because they lead to acquiring other resources (time, knowledge, relations) [14]. In the current study, "objects" were assessed through residential property since many studies have provided evidence for the relationship found between poor housing conditions and poor health [15]. Moreover, according to Maslow's [16, 17] hierarchy of needs, such physiological needs are the basic needs people are motivated to achieve before proceeding on to fulfil the next hierarchy of needs. Regarding "condition", we chose gender, health state and witness to injury to represent this class, since these variables are known to have an impact on people’s vulnerability to adverse effects of trauma, as well as the way people react to such impact [18, 19]. With relation to "energy" class, social support and the quality of social interaction are known to play an important role in predicting PTSD symptoms in the aftermath of a traumatic event [20]. Moreover, according to Siedlecki et al. [21] different facets of subjective well-being were found to be predicted by different aspects of social support (namely, perceived support, enacted support, and family embeddedness). Therefore, relationships were chosen to represent this class. Finally, regarding "personal" class, coping strategies were chosen since their link with psychological well-being is well documented [22].

Underlying Conservation of Resources theory is the understanding that people strive to attain and protect their resources at all times. Stress is experienced when these resources are perceived to be threatened, or actually used, spent, or otherwise lost [12]. An extensive body of research supports the notion that loss of resources predicts psychological distress following various traumatic experiences, including floods, hurricanes, earthquakes, terror attacks, and mass shootings (e.g., [14, 23–26]). Thus, it is anticipated that a person’s well-being will be influenced by his/her available resources depending on the value that he/she has associated with the particular resources that are threatened.

In sum, the present study aims to evaluate subjective well-being following Typhoon Haiyan and its association with four broad classes of resources offered by the Conservation of Resources theory namely: object—residential property; condition—gender, witness to injury and health state; personal—coping strategies; energy–relationships.

## Methods

### Ethics statement

The study was conducted in accordance with the ethical standards and approved by the Institutional Review Board of the School of Social Work at Ariel University. Participation was voluntary. Questionnaires and a project description were distributed by Asia Opinions LTD panel company (http://www.asiaopinions.com). To recruit the study participants, Asia Opinions sent e-mails to invite potential participants from their databases to enroll in the current study following a screening process to ensure eligibility (based on age, place of residence and current Filipino level of fluency). All participants indicated agreement to participate by completing the survey anonymously.

### Participants and procedure

Following approval by the Institutional Review Board, an online survey was conducted in the Philippines within 3 weeks of the November 3, 2013, the date of the typhoon (i.e. the week of 27.11.13 on). Inclusion criteria were age 18 or older, belonging to areas affected by typhoon Haiyan. One thousand participants gave their informed consent to complete a self-report questionnaire written in Filipino (response rate was 83.3%, out of a potential 1,200) (data in S1 Text).

Rate of missing values in the research was between 0.1% and 7% per item. In order to test if we can assume that missing values are Missing Completely at Random (MCAR), the Little test was performed [27]. Test results allowed us to assume that missing values are indeed at random (χ^2^_(2379)_ = 2262.546, p = 0.95). Using listwise deletion and excluding all subjects with missing values as recommended by Alison [28] led to exclusion of 167 (16.68%) respondents. Sample size for all analyses was N = 834, 50% female, M age 30.34 (SD 10.28). Compared to census information from two separate sources [29, 30] indicating 49.8% of male representation and median age of 23, it appears that the study’s sample of 18-year-old and older is slightly skewed towards older and more educated population than the Philippine’s population.

### Measures

Participants filled out self-report measures using an online interview system regarding: a) socio demographic data: age, marital status, education, self-rated health; b) subjective well-being perception via the Delighted Terrible Faces Scale (DTS), a bi-dimensional, single item scale that measures subjective well-being [31, 32] using seven response categories, ranging from terrible (1) to delighted (7); c) disaster related experiences: Three yes/no items asking about personal property loss, witnessing injury, and damage to home; d) Coping strategies: Three subscales from the Craver’s Brief Cope Inventory [33] were also included: Self Blame (α = 0.72), Active Coping (α = 0.86), and Reframing (α = 0.820). These coping strategies were chosen in line with Adams et al's [34] results that point to the importance of emotion-focused coping strategies (e.g., sharing of feelings, minimization of the situation, distraction) over problem focused coping strategies(e.g., planning, direct action) in the face of the unpredictable and massive nature of the Katrina disaster; e) Personal relationships obtained through support sources: close family, relatives and friends, community (yes/no) and assessing problems with those relationships after Haiyan (α = .77).

### Data analysis

All analyses were done using IBM SPSS Statistics 20 (IBM Corp 2011) [35]. Scales were tested for normality using skewness and kurtosis calculations. Maximum absolute skewness was 1.03 (for Positive reframing scale) and maximum absolute kurtosis was 0.65 (for Self-Rated Health) (See Table 1). Pearson correlations were appropriate indicators of the association between the four classes of resources object -residential property; condition -gender, health state and witness to injury; personal -coping strategies; energy–relationships and the dependent variable—subjective well-being.

**Table 1:** Mean, SD, skewness and kurtosis for research variables.

Variable	Mean	SD	Skewness	Kurtosis
Delighted-Terrible Scale	4.77	1.16	-0.05	0.45
Self-Rated Health	2.88	0.62	-0.38	0.65
Problems in relations	16.31	3.52	-0.01	-0.15
Active coping	6.40	1.59	-0.88	0.16
Positive reframing	6.44	1.57	-1.03	0.58
Self-blame	4.42	1.61	0.33	-0.46

Four hierarchical regression analyses were performed to ascertain the unique and cumulative contributions of the independent variables to the explanation of the variance in subjective well-being (measured by the Delighted Terrible Faces Scale—DTS). Objective resources were entered in the first step, while condition resources entered in the second step. The personal resources came in the third step and energy resources were entered in the fourth step. This sequence stems from Maslow's hierarchy of needs [36] which suggests that the most basic level of needs must be met before the individual will strongly desire (or focus motivation upon) secondary or higher-level needs. A p-value of less than 0.05 was considered statistically significant. Regression model was tested for collinearity (minimum tolerance value was 0.534) and for correlation of residuals (Durbin-Watson = 2.04), indicating low collinearity and no autocorrelation. In addition, models which included curvilinear effects as well as interaction affects between tangible and nontangible losses of resources were also tested. Results indicated that adding these elements to the models did not improved the rate of explained variance of subjective well-being. Using the HC3 heteroskedasticity-consistent standard error estimator [37] for the full model revealed similar results to the OLS regression, implying the same variance across all observation points.

## Results

### Correlations between the four resource clusters and subjective well-being

Pearson correlations carried out between the clusters of object, condition, personal and energy resources on the one hand and subjective wellbeing on the other, yielded very low associations for all four resource clusters (less than 0.3), although significant as shown in Table 2.

**Table 2:** Pearson correlations between research variables.

	1	2	3	4	5	6	7	8	9	10
1. Delighted-Terrible Scale										
2. Property Loss	-.013									
3. Home Damaged	-.063*	.580***								
4. Witness Injury	-.066*	.234***	.186***							
5. Gender (female)	.067*	-.117***	-.106**	-.135***						
6. Self-Rated Health	.236***	.036	.021	.081**	-.061*					
7. Active Coping	.152***	-.044	.000	.026	.093**	.114***				
8. Positive Reframing	.170***	-.058*	-.027	-.023	.108***	.085**	.675***			
9. Self-Blame	-.215***	-.030	.017	.071*	-.057*	-.133***	.133***	.113***		
10. Relations	.118***	.013	.068*	.005	.025	-.014	.058*	.066*	-.072*	
11. Problems in relations	-.162***	-.129***	-.132***	-.147***	-.001	-.186***	-.126***	-.142***	-.017	.021

*** p<0.001,

** p<0.01,

* p<0.05

### Contributions to subjective wellbeing

Hierarchical regression analyses were performed to ascertain the unique and cumulative contributions of the independent variables for explaining variance of subjective well-being. Table 3 presents standardized coefficient regression in predicting subjective wellbeing.

**Table 3:** Standardized coefficients of OLS regression on Delighted-Terrible Scale.

	Model 1			Model 2			Model 3			Model 4		
	B	β	t	B	β	t	B	β	t	B	β	t
Property Loss	.156	.036	0.84	.203	.047	1.12	.188	.043	1.07	.169	.039	0.97
Home Damaged	-.344	-.084	-1.98*	-.305	-.075	-1.81	-.291	-.071	-1.78	-.372	-.091	-2.29*
Witness Injury				-.187	-.073	-2.11*	-.149	-.058	-1.72	-.187	-.073	-2.18*
Gender (female)				.162	.070	2.06*	.086	.037	1.12	.075	.032	0.98
Self-Rated Health				.456	.246	7.31***	.365	.197	5.92***	.332	.179	5.38***
Active Coping							.055	.075	1.70	.050	.069	1.57
Positive Reframing							.089	.121	2.74**	.074	.100	2.29*
Self-Blame							-.147	-.204	-6.11***	-.142	-.197	-5.95***
Relation										.247	.103	3.20**
Problems in Relations										-.043	-.129	-3.87***
Adjusted R Square	.002			.066			.124			.147		
F	2.03			12.79***			15.78***			15.40***		
Δ R Square	.005			.067			.061			.025		
F Change	2.03			19.88***			19.33***			12.17***		

*** p<0.001,

** p<0.01,

* p<0.05

As can be seen, when the resources for the *objects* cluster were entered first, we found no significant contribution to the explained variance of subjective well-being. In the second step with regard to the clusters of *condition* resources, the explained variance was statistically significant explaining 6.6% of the variation (with contribution of 6.7% for this step). The variables that account for this contribution were witness to injury, female gender and self-rated health. This is to say that women survivors and those who rated their health as good exhibited higher subjective well-being, while those survivors who witnessed injury reported less subjective well-being.

With the third step, when the *personal* resource was entered, the explained variance of subjective well-being was 12.4%. Positive reframing and self-blame contributed to the explained variance of subjective well-being (contribution of 6.1% by this step). That is, survivors who were engaged with positive reframing and those who used less self-blame as a way of coping showed higher subjective well-being. At this step, only self-rated health remained significant (condition resources). For the last step, the clusters of *energies* resources were entered with a contribution of 14.7% to the explained variance of subjective well-being. Both relations and problems in relations were significant and accounted for the variance of subjective well-being (contribution of 2.5%). Hence, those who reported having social relations and those who noted fewer problems in relations revealed higher subjective well-being. At this step, the personal resources cluster—as well as self-rated health and having witnessed injury continued to be significant. In addition, home damage (object resources) re-emerged.

## Discussion

Our study focused on subjective well-being following the Super Typhoon Haiyan (traumatic event) during the period that was identified with a potential acute stress disorder (ASD). The current findings show that subjective well-being can be predicted by various resources; objects, personal characteristics, conditions, and energies. That is to say that individuals' resources might predict their subjective well-being following natural disasters, because any loss of these resources, whether actual or perceived, or failure to gain resources as expected, causes psychological distress [14]. Our findings are consistent with literature documenting the application of Conservation of Resources theory in natural disaster scenarios [25, 38–41].

Closer observation of the components of those resources revealed interesting findings. With regard to the cluster of *objects* it seems that home damaged contributed to subjective well-being, but not property loss. This result confirmed previous findings which indicated that residential damage was found to have long-term negative impact on emotional well-being among Hurricane Katrina's survivors [42]. Nevertheless, it contributed to subjective well-being, only when it entered with all of the other resource clusters. This may be explained according to Maslow's hierarchy of needs [16,17], that stated that an individual is ready to act upon the growth needs if and only when the deficiency needs are met.

With respect to *condition* resources, self-rated health was the dominant factor in predicting subjective well-being. As noted previously by Diener et al., [43] health plays a large role in determining life satisfaction, thus, it can be assumed that when survivors of natural disasters have poor estimations of their health, it may be more effective to focus on improving the conditions of their subjective health even when objective measures such as a physician’s observations and diagnoses indicate better health.

In relation to *personal characteristic* resources, positive reframing and self-blame served as possible predictors of subjective well-being (they were found significant predictors, but explained a small amount of the variation). According to Linley and Joseph [44] cognitive processing is necessary for the rebuilding of shattered worldviews following trauma. Thereby, those survivors who used a positive reframing manner of coping revealed higher subjective well-being. Regarding the self-blame way of coping, since it can lead to feelings of worthlessness and low self-esteem [45], it is reasonable to conclude that using this type of coping might predict low subjective well-being. Interestingly, active coping had no significant effect on subjective well-being in the regression. This finding is in line with Adams et al.'s [34] results among police officers who served as first responders to the Katrina disaster. It has been argued that problem-focused coping predominates when individuals believe that something can be done about a situation, whereas emotion-focused coping predominates when it is believed that the crisis has to be endured [46]. Thus, it might be that the erratic and dynamic nature of the disaster hindered the ability of the survivors to rely on problem-focused coping practices because they did not have the luxury of relying on normal modes of communication and functional equipment as during routine times.

Finally, with respect to the *energies resource* cluster, our results indicated that having social relations and perceived problems with relations were found to predict subjective well-being. These results are in line with previous studies that showed that social support increases well-being and limits distress after mass trauma [25, 47–49]. Being connected to others makes it easier to obtain knowledge needed for disaster recovery, to find practical help for solving problems, to gain a sense of being understood, and to share of trauma experiences and tips about coping. However, when social relations are perceived to be negative, subjective well-being declined. This result is in line with Seppala et al.'s [50] who stated that, individuals with satisfying social relationships reported above-average levels of happiness, lower levels of depression and anxiety, and higher resiliency across a broad array of stressful life events and environments.

Several important limitations should be noted. First, since the study’s participants were older and more educated than the Philippines census, caution should be applied concerning the generalization of the findings. Second, we cannot rule out that this particular survey did not adequately capture the Haiyan–exposed population (for example people who didn't have access to the Internet due to property damage). Third, the self-report design of this study and its cross-sectional assessments, had neither baseline measures nor long-term outcomes. Fourth, measurement of coping was limited, since the primary aim of this study was to determine factors leading to subjective well-being. Thus, we cannot rule out that other cultural ways of coping could have been utilized by the people living in the Philippines. Fifth, since single-items measure of subjective well-being cannot cover all aspects of well-being, multi-item scales of subjective well-being are required [51]. It is recommended that future studies follow participants longitudinally, and seek positive reactions to traumatic events alongside negative reactions, soon after the event and after an extended period of time. That type of assessment will help to understand predictors of subjective well-being over time following natural disasters, along with the relationship of subjective well-being to acute stress disorder. Furthermore, it would be interesting to investigate the relationship between time elapsed from the disaster until participants answered the survey (by tracking the dates that the questionnaires were answered online). Such a design might contribute to our understanding of the psychological reactions that accompany exposure to natural disasters across time, and a better understanding of self-report design in disaster settings.

In addition, it is recommended to further examine resources that were not found to be predictive of subjective well-being (i.e. property loss, gender—being a female and active coping).

## Conclusions

Our findings highlight the need to strengthen and protect the pool of individual resources in the wake of traumatic events–such as natural disaster, in order to promote positive outcomes in terms of subjective well-being. Interventions that focus on restoring psychosocial resources, in addition to restoring tangible possessions, could improve the subjective well-being of survivors of natural disasters.

## Supporting information

S1 Text Supertyphoon_Meni 23 11 13 Wave one. (DOCX)
