# Supplementary material for: Typhoon survivors' subjective wellbeing—A different view of responses to natural disaster
Source: PLoS One. 2017 Sep 6;12(9):e0184327. doi: 10.1371/journal.pone.0184327 (PMC5587279; doi:10.1371/journal.pone.0184327)
Supplement: S1 Text — (DOCX) [file pone.0184327.s001.docx]

Demographics (Age, Gender, marital status, years of education, income, religiosity) will be provided by the survey company.

Part I

What is your marital status?

1. Married or living together with a spouse (partner).
2. Single or never married.
3. Divorced or separated.
4. Widowed.

What is your religiosity level?

1. Not religious.
2. A little religious.
3. Moderately religious.
4. Very religious.

1. "How do you feel about your life as a whole.....?" Delighted-Terrible scale [well-being]

1. Terrible
2. Unhappy
3. Mostly dissatisfied
4. Mixed
5. Mostly satisfied
6. Pleased
7. Delighted

2. Please rate the following statement “I am afraid of death” Death Anxiety

1. Strongly disagree.
2. Disagree.
3. Disagree a little.
4. Nor agree or disagree.
5. Agree a little
6. Agree
7. Strongly agree.

3. “How do you rate your health”?

1. Bad.
2. So-so.
3. Good.
4. Excellent.

Questions 4-5, Cognitive bias.

4. A fair coin turns up *Heads* 5 times in a row. Which is more likely to happen on the next toss?

1. Heads.
2. Tails.
3. Neither – chance is 50-50.

5. When I was a kid, my best friend was 1 of 3 kids in the family. All 3 kids were boys.

Then their mom got pregnant. The mom said, “Since I have 3 boys, I’ll probably have a girl.”

What will she have?

1. Boy.
2. Girl.
3. Neither – chance is 50-50.

Use of Media

Part II

| 6. What was your source of information regarding the super Typhoon Haiyan? | No | Yes |
| --- | --- | --- |
| 6a.TV | 0 | 1 |
| 6b. Radio | 0 | 1 |
| 6c. Newspaper | 0 | 1 |
| 6d. Internet | 0 | 1 |
| 6e. Social Network (FACEBOOK) | 0 | 1 |
| 6f. News Websites | 0 | 1 |
| 6g. Twitter | 0 | 1 |
| 6h. Youtube | 0 | 1 |

Coping three sub-scales taken from Craver’s Brief Cope

Part III

| Try to rate each item separately in your mind from the others. Make your answers as true FOR YOU as you can. | | | | |
| --- | --- | --- | --- | --- |
|  | I haven't been doing this at all | I've been doing this a little bit | I've been doing this a medium amount | I've been doing this a lot |
| 7. I've been concentrating my efforts on doing something about the situation I'm in. | 1 | 2 | 3 | 4 |
| 8. I've been taking action to try to make the situation better. | 1 | 2 | 3 | 4 |
| 9. I've been trying to see it in a different light, to make it seem more positive. | 1 | 2 | 3 | 4 |
| 10. I’ve been criticizing myself. | 1 | 2 | 3 | 4 |
| 11. I've been looking for something good in what is happening. | 1 | 2 | 3 | 4 |
| 12. I’ve been blaming myself for things that happened | 1 | 2 | 3 | 4 |

Part IV

Disaster related experiences

|  | No | Yes |
| --- | --- | --- |
| 13. Did you lose personal property or belongings during the super Typhoon Haiyan | 0 | 1 |
| 14. Did you witness an injury during the super Typhoon Haiyan | 0 | 1 |
| 15. Did your home was damaged during the super Typhoon Haiyan | 0 | 1 |

Part V

Meaning in life and flourishing (Kraue, 2004).

| Please rate the following statement: | | | | |
| --- | --- | --- | --- | --- |
|  | agree strongly | agree somewhat | disagree somewhat | disagree strongly |
| 16. I have a system of values and beliefs that guide my daily activities. | 1 | 2 | 3 | 4 |
| 17. I have a philosophy of life that helps me understand who I am. | 1 | 2 | 3 | 4 |
| 19. I feel like I have found a really significant meaning in my life. | 1 | 2 | 3 | 4 |
| 20. The world is a dangerous place. |  |  |  |  |
| 21. I have a sense of direction and purpose in life | 1 | 2 | 3 | 4 |

K6 (Kessler et al., 2003) – Psychological distress

Part VI

| Please circle the number that best describes how often you had this feeling since the super typhoon Haiyan | | | | | |
| --- | --- | --- | --- | --- | --- |
|  | All of the Time | Most of the Time | Some of the Time | A little of the Time | None of the Time |
| 22. Nervous | 1 | 2 | 3 | 4 | 5 |
| 23. Hopeless | 1 | 2 | 3 | 4 | 5 |
| 24. Restless or fidgety | 1 | 2 | 3 | 4 | 5 |
| 25. So depressed that nothing could cheer you up | 1 | 2 | 3 | 4 | 5 |
| 26. That everything was an effort | 1 | 2 | 3 | 4 | 5 |
| 27. Worthless | 1 | 2 | 3 | 4 | 5 |

Relationships

Part VII

Questions for follow up Super Typhoon Haiyan

28. Are you currently in a romantic relationship/ married? If Yes then answer the following

1a. How satisfied are you in your relationship (1 - very unsatisfied, 2 - a little unsatisfied,3 - neither satisfied or unsatisfied, 4 - a little satisfied, 5- very satisfied)

1b. How has your relationship changed since the typhoon? (1 - closer than before, 2 - about the same as before, 3 - less close than before)

1c. Have you had any arguments with your partner as a result of the typhoon? (1 - yes, many arguments; 2 - a few arguments; 3 - no arguments)

1d. How committed are you to your relationship (1) completely committed; I would do anything to maintain this (2) quite committed; I would make an effort to maintain this (3) neither committed or uncommitted (4) Not really committed

1e. Has your relationship commitment changed since the typhoon (1 - more committed than before, 2- about the same level of commitment, 3 - less commitment than before)

29. How close are you to your family members (immediate family members such as parents, grandparents, siblings) (1 - very close, 2 - quite close, 3- only a little close, 4- quite distant, 5- very distant)

30. How close do you feel you have been to your family members (immediate family members such as parents, grandparents, siblings) since the typhoon? (1 - Closer than before, 2- about the same as before, 3 - less close than before, 4- don’t know)

31. Thinking about those in your neighborhood (acquaintances, neighbors etc. but not people you know well), how close are you to them? (1 - very close, 2 - quite close, 3 - only a little close, 4 - quite distant, 5- very distant)

32. Since the typhoon, how have you felt towards them? (1 - Closer than before, 2 - about the same as before, 3 - less close than before, 4 - don’t know)

33. How do you think the typhoon changed relationships with your acquaintances and neighbors? (1. More united, with prior disagreements more likely to disappear, 2. There have been no real changes since the typhoon, 3. People are now less united and more likely to disagree since the typhoon 4. don’t know)

34. What about these acquaintances’ openness, honesty, and sincerity since the typhoon? (1. People are more open, sincere, and honest towards each other, 2. People are about the same as they were before the typhoon, 3. People are less open, sincere and honest than they were before the typhoon 4. don’t know)

35. Now I want you to think about other Philipinnos OUTSIDE your community. How close do you feel to them? (1 - very close, 2 - quite close, 3 - only a little close, 4 - quite distant, 5 - very distant, 6 - don’t know)

36. Since the typhoon how have you felt towards those outside your community? (1. Closer / more intimate than usual 2. The same as usual 3. More distant / less intimate than usual 4. don’t know)

37. How do you think the typhoon changed relationships with those from outside the community? (1. More united, with prior disagreements more likely to disappear, 2. There have been no real changes since the typhoon, 3. People are now less united and more likely to disagree since the typhoon 4. don’t know)

Part VIII

Please read each one carefully, put an answer whether you have experience the following and to what extent since super Typhoon Haiyan.

DSM 5 ASD Criteria

*Please use the following Categories.*

| **Not at all (1)** | **A little bit (2)** | **Moderately (3)** | **Quite a bit (4)** | **Extremely (5)** |
| --- | --- | --- | --- | --- |

| Experience and behaviors presented following Super Typhoon Haiyan | Did you experience the following | Response Category | | | | |
| --- | --- | --- | --- | --- | --- | --- |
| 38. A. Exposure to actual or threatened death, serious injury in one (or more) of the following ways:  1. Directly experiencing the Super Typhoon Haiyan  2. Witnessing, in person, the Super Typhoon Haiyan as it occurred to others.  3. Learning that the Super Typhoon Haiyan occurred to a close family member or close friend. | Yes/No | 1 | 2 | 3 | 4 | 5 |
| 39. B1. Recurrent, involuntary, and intrusive distressing memories of the Super Typhoon Haiyan | Yes/No | 1 | 2 | 3 | 4 | 5 |
| 40. B2. Recurrent distressing dreams in which the content and/or affect of the dream are related to the Super Typhoon Haiyan | Yes/No | 1 | 2 | 3 | 4 | 5 |
| 41. B3. Dissociative reactions (e.g., flashbacks) in which the individual feels or acts as if the Super Typhoon Haiyan were recurring. | Yes/No | 1 | 2 | 3 | 4 | 5 |
| 42. B4. Intense or prolonged psychological distress or marked physiological reactions in response to internal or external cues that symbolize or resemble an aspect of the Super Typhoon Haiyan | Yes/No | 1 | 2 | 3 | 4 | 5 |
| 43. C1. Persistent inability to experience positive emotions (e.g., inability to experience happiness, satisfaction, or loving feelings) following the Super Typhoon Haiyan. | Yes/No | 1 | 2 | 3 | 4 | 5 |
| 44. D1. 6. An altered sense of the reality of one’s surroundings or oneself (e.g., seeing oneself from another’s perspective, being in a daze, time slowing) following the Super Typhoon Haiyan. | Yes/No | 1 | 2 | 3 | 4 | 5 |
| 45. D2. Inability to remember an important aspect of the Super Typhoon Haiyan | Yes/No | 1 | 2 | 3 | 4 | 5 |
| 46. E1. Efforts to avoid distressing memories, thoughts, or feelings about or closely associated with the Super Typhoon Haiyan | Yes/No | 1 | 2 | 3 | 4 | 5 |
| 47. E2. Efforts to avoid external reminders (people, places, conversations, activities, objects, situations) that arouse distressing memories, thoughts, or feelings about or closely associated with the Super Typhoon Haiyan | Yes/No | 1 | 2 | 3 | 4 | 5 |
| 48. F1. Sleep disturbance (e.g., difficulty falling or staying asleep, restless sleep). | Yes/No | 1 | 2 | 3 | 4 | 5 |
| 49. F2. Irritable behavior and angry outbursts (with little or no provocation), typically expressed as verbal or physical aggression toward people or objects. | Yes/No | 1 | 2 | 3 | 4 | 5 |
| 50. F3. Hypervigilance (highly alert) | Yes/No | 1 | 2 | 3 | 4 | 5 |
| 51. F4. Problems with concentration | Yes/No | 1 | 2 | 3 | 4 | 5 |
| 52. F5. Exaggerated startle response. | Yes/No | 1 | 2 | 3 | 4 | 5 |
| 53. G1. Difficulty to function or significant distress or impairment in social, occupational, or other important areas of functioning following Super Typhoon Haiyan | Yes/No | 1 | 2 | 3 | 4 | 5 |
